# Smartphones dependency risk analysis using machine-learning predictive models

**DOI:** 10.1038/s41598-022-26336-2

**Published:** 2022-12-31

**Authors:** Claudia Fernanda Giraldo-Jiménez, Javier Gaviria-Chavarro, Milton Sarria-Paja, Leonardo Antonio Bermeo Varón, John Jairo Villarejo-Mayor, André Luiz Felix Rodacki

**Affiliations:** 1 Department of Health, Universidad Santiago de Cali, Cali, Colombia; 2 Doctoral Program in Applied Sciences, Universidad Santiago de Cali, Cali, Colombia; 3 Department of Engineering, Universidad Santiago de Cali, Cali, Colombia; 4 Department of Electrical and Electronic Engineering, Federal University of Santa Catarina, Florianopolis, Brazil; 5 Department of Physical Education, Federal University of Paraná, Curitiba, Paraná Brazil

**Keywords:** Occupational health, Electrical and electronic engineering, Risk factors

## Abstract

Recent technological advances have changed how people interact, run businesses, learn, and use their free time. The advantages and facilities provided by electronic devices have played a major role. On the other hand, extensive use of such technology also has adverse effects on several aspects of human life (e.g., the development of societal sedentary lifestyles and new addictions). Smartphone dependency is new addiction that primarily affects the young population. The consequences may negatively impact mental and physical health (e.g., lack of attention or local pain). Health professionals rely on self-reported subjective information to assess the dependency level, requiring specialists' opinions to diagnose such a dependency. This study proposes a data-driven prediction model for smartphone dependency based on machine learning techniques using an analytical retrospective case–control approach. Different classification methods were applied, including classical and modern machine learning models. Students from a private university in Cali—Colombia (n = 1228) were tested for (i) smartphone dependency, (ii) musculoskeletal symptoms, and (iii) the Risk Factors Questionnaire. Random forest, logistic regression, and support vector machine-based classifiers exhibited the highest prediction accuracy, 76–77%, for smartphone dependency, estimated through the stratified-k-fold cross-validation technique. Results showed that self-reported information provides insight into predicting smartphone dependency correctly. Such an approach opens doors for future research aiming to include objective measures to increase accuracy and help to reduce the negative consequences of this new addiction form.

## Introduction

The use of smartphones and mobile devices has experienced exponential growth in the last decade, and such devices have become usual for work, education, daily tasks, and social life^1^. An added value of smartphones is based on personal use for everyday organization, communication, and entertainment, increasing the ubiquity of digital tools during daily routines^2^. Despite many positive aspects, some adverse effects are derived from extensive usage by young individuals^3,4^. The use of mobile devices is an occupational reality^5^. The impact of smartphone usage on cognitive abilities for educational, occupational, and social functioning can be classified as negative or positive from their socio-emotional components^6^. Moreover, the impact on children and teenagers' physical and mental health has been evidenced, modulated by exposure times and compulsive behaviors^7^. Smartphones have a repertoire of tools that have altered consumption dynamics and how users interact within different environments^8^. There are many instances where other organizations (e.g., industrial, educational, commercial, and advertising sectors) have created mobile applications for communication purposes^9^. These applications help to improve collaboration and facilitate information exchange^10^. They also provide the business sector with information on the improvement and development of mobile applications to achieve business objectives, cover new markets, and attract demand^11^. Available tools have three main features: accessibility, repetition, and interactivity, which generate a high affinity towards these devices, whereas smartphones stand out^12^. Consumer and information applications and social networks have high demand and influence individual communication and lifestyle habits^13^.

Over the last decade, the use of mobile devices in different communities has become widespread, and its lasting effects have multiplied. For instance, smartphones are effective learning tools in educational settings to gain knowledge. There is a significant effect on the academic performance of undergraduate students when mobile applications intermediate learning compared to traditional learning schemes^14^. In this way, technological improvements in the educational sector create the need to propose new strategies to offer students guidance using efficient management of technical resources, to strengthen the learning process^15^. The excessive use of smartphones is more prevalent among student populations than others. Easy access to the internet and big screens for game interaction are factors significantly associated with blindness, deafness, and inattentiveness^23^.

Mobile device dependency is a problem established in terms of frequency and excessive use. There is a prevalence of approximately 40% excessive use of mobile devices overall users, and about 42% of them belong to the group of middle-low-class households^16^, with significant representation in the young population^17^. This habit is negatively associated with inhibition, decision-making, memory performance, and sleep disorders^18^. Besides, the simultaneous use of a cell phone during daily activities may represent an overload for some muscle groups and constitute a risk factor for musculoskeletal onset problems^19^.

Different studies on the problems derived from the use of mobile devices show preferences toward gaming. However, users do not use these devices for gaming purposes but also multiple-purpose applications ^20^. These applications are an integral part of modern life and, therefore, can create adverse dependency effects^21^. Consequently, it is crucial to quantify the dependency using accurate scales and to incorporate ways of analyzing the effects of excessive and harmful smartphone use^22^.

Implementing strategies to detect and monitor risk factors associated with smartphone dependency is imperative. These strategies should promote participation in recreational activities and strengthen social relationships. Reducing the adverse effects of smartphone dependency, postural problems, musculoskeletal symptoms, and even deformities or chronic injuries may be prevented. The negative consequences on academic performance, working, and social life can also be influenced.

In this study, the Smartphone Dependency Test (SDT) questionnaire was used to assess dependency among university students. The SDT questionnaire was validated and linguistically adapted in 2016 for public and private university students, with reliability for abstinence and tolerance (α = 0.901), for abuse and difficulty in controlling the impulse (α = 0.853), and for problems caused by excessive use (α = 0.762)^24^.

Research involving predictive models to assess smartphone dependency is scarce. To the best of our knowledge, there are no studies quantifying and using analytical techniques such as machine learning to model variables associated with smartphone dependency. It is worth mentioning that there is a significant advance in using these tools to solve different research problems^25,26^. However, they have not been widely used to generate predictive models focused on smartphone dependence and have not been established^27,28^.

This study proposed using self-reported information gathered through standardized questionnaires to train predictive models using a machine-learning approach. It was hypothesized that the proposed questionnaires could help to encode self-reported subjective information, which can be used to predict smartphone dependency. The input variables consider factors related to personal data, family, environmental risks, physical loading, device-specific risk factors, and musculoskeletal symptoms. Such an approach may reduce the bias during the assessment process. This also may assist professionals in recommending actions to reduce the adverse effects of overusing mobile devices. To our knowledge, no previous studies address this issue from a data-driven models’ standpoint. This study also provides insights that may entitle one to link subjective cues to objective measures in future analyses.

## Methods

### Participants and procedures

The study is an analytical observation using a retrospective case–control approach involving 14,858 students from 19 undergraduate programs. The students were registered in four schools of a private university in Cali, Colombia, in 2019. A 95% confidence level and a 5% margin of error were used for the sample calculation, resulting in a sample of 1247 students. The sampling technique was randomly stratified. The selection of the participants was performed by probability sampling using the epi-info™ suite^29^. Eighteen individuals were excluded after they met the exclusion criteria. These participants used the upper limbs (arms and hands) in regular physical activities such as high-impact sports (basketball, volleyball, table tennis, and weights in the gym) and repetitive movement in artistic activities (such as painting, embroidery crafts, and playing musical instruments such as guitar and drum).

Consequently, the frequency and intensity of these activities could cause information bias, allowing the control for selection bias. Therefore, the final sample was recalculated for 1228 students (95% CI; 5% error). The Levene test confirmed data homogeneity, and the sample was comparable in age, sex, program, semester, and marital status (0.157–0.740). The participants were then assigned according to their smartphone dependency. The case group was composed of students with some smartphone dependency, while the control group was formed of students with no smartphone dependency.

The volunteers signed an informed consent form before participating in the study. Those individuals who submitted an incomplete form or frequently played sports or artistic activities involving the upper limbs were excluded.

The Smartphone Dependency Test is a free-to-use test created by Chóliz^30^, which was validated and linguistically adapted in 2016 for students receiving both public and private education^31^. This test was used to measure the level of independence of Mobile Devices (MD), which was assigned as the dependent variable. The test lasted 10 min and consisted of 22 items presented using a Likert-type scale. The scores range from 0 (zero) to 88 as the maximum to determine whether the dependency was absent (0–29), low (30–38), medium (39–48), or high (49–88). In addition, musculoskeletal disorders (MSD) were characterized via the Nordic Questionnaire, in its Spanish version, whose application lasted 7 min. The questionnaire comprised two levels: (i) a general level that sought to determine the occurrence of musculoskeletal discomfort by anatomical regions, and (ii) a specific level that focused on delving into the chronology, frequency, duration, intensity, and impact of the discomfort on their everyday activities.

The risk factors were the independent variables. The Risk Factors Questionnaire was designed and subjected to internal validation by the researchers through the Delphi method by a group of 6 experts, obtaining a validity of approximately 0.9, according to Chronbach's alpha; its application lasted 7 min. This questionnaire included the variables considered in the theoretical framework about sociodemographic, interpersonal, and contextual factors related to the device and physical load. It was possible to identify the risk factors in the university student population^32^.

The study followed the principles of the Helsinki Declaration, guaranteeing confidentiality by coding and signing the informed consent before participation. Regarding data collection, this study protocol was doubly reviewed and endorsed by the Scientific Committee of Ethics and Bioethics of the Universidad Santiago de Cali (act # 03 of 2019).

### Data analysis

The data were recorded by a double entry in Excel. The information from the two databases was compared, and unmatched data were cleaned, performing verification in the primary source.

To structure the model construction, the variables were transformed into categorical types for the processing and analysis phase. The data allocation, which was 1%, was performed using the mode for qualitative variables and the arithmetic mean for quantitative variables. Once the information was validated, a descriptive exploratory analysis of the different variables was conducted to determine their behavior. Subsequently, a bivariate analysis was performed to determine which were included in the model and selected for statistical significance with a *p*-value < 0.05.

**Figure 1:**
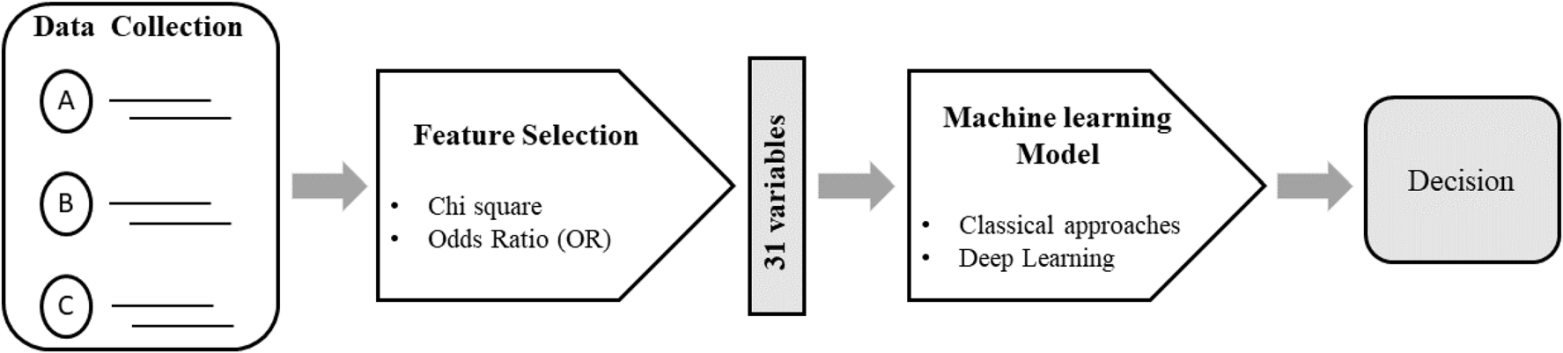
Automatic system for predicting smartphone dependency.

Figure 1 shows a schematic representation of the research approach. It indicates a general-purpose pattern-recognition system adapted to address the overuse of smartphones. First, participants answered three questionnaires (i.e., the Smartphone Dependency Test, the Nordic Questionnaire—Spanish version, and the Risk Factors Questionnaire) used by health professionals to assess the participant dependency level. Next, a selection strategy and descriptive exploratory analyses of the different variables were performed to determine which predictors were highly correlated to the target variables. As a result, 31 variables were selected and used to feed the data-driven predictive model. Two groups of algorithms were applied—i.e., the classical approach and the deep learning approach. The details of the algorithms are provided in the following section. Finally, based on these predictive models, smartphone dependency and overusing were estimated.

**Figure 2:**
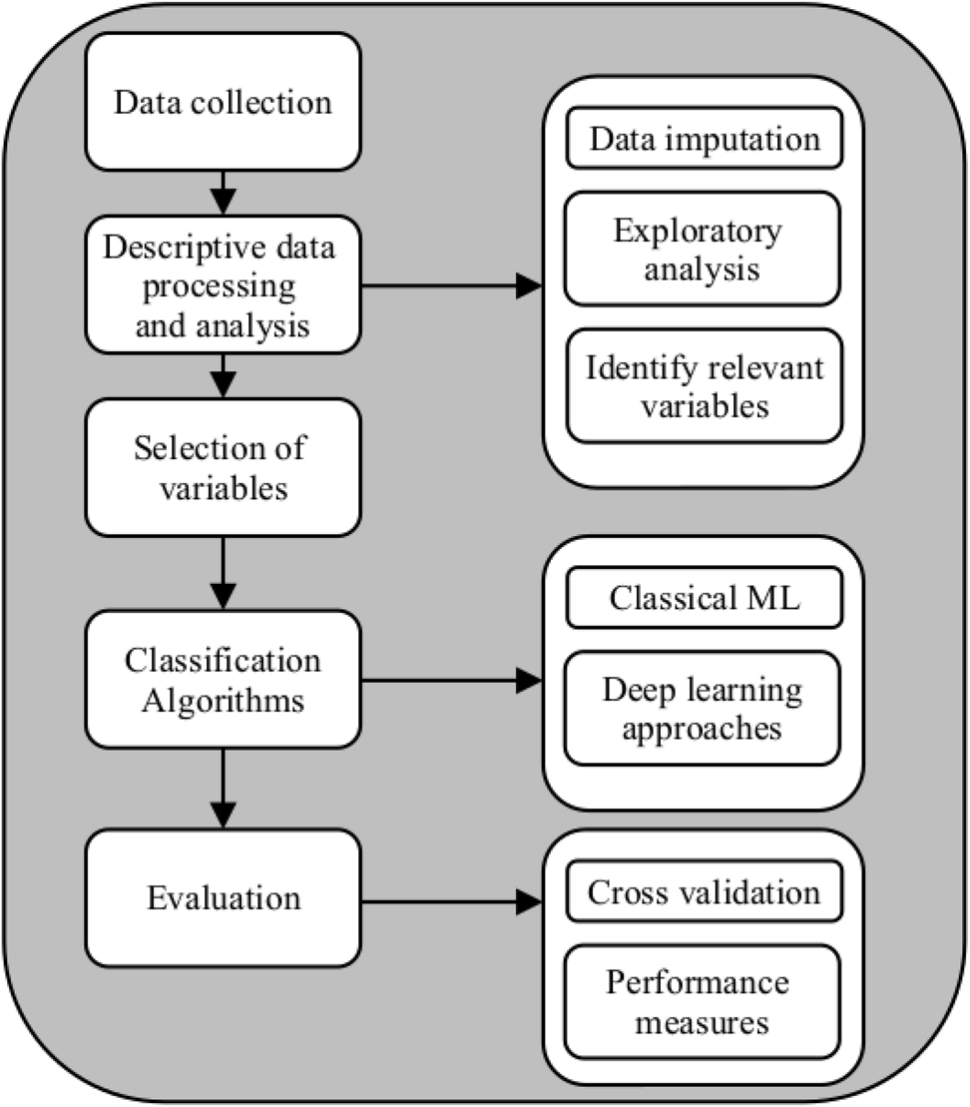
Information processing flowchart to find out the model.

Data processing, debugging, modeling, and validation were structured in six stages and are described in Fig. 2.

### Supervised machine learning techniques

Machine learning has been successfully used in several research areas with applications in medical signal processing, computer-assisted systems, language processing, and healthcare, among others. From the classical point of view to more recent deep learning techniques, data-driven models try to capture the inner structure of data derived from external systems. These models help make predictions on new unseen data^26,33,34^. There is a wide range of applications that vary from healthcare, transportation, social networks, banking, security, and education. Internet of Things (IoT) Networks is widespread in many industrial applications. Machine Learning models help identify and avoid malicious traffic attacks, which can affect network security and essential services^35–38^. These techniques have been used to improve the user’s experience and decision-making process, which are more subjective scenarios and more dependent on the user’s psychological characteristics^39,40^. It is important to note that in such scenarios, it is necessary to analyze people’s opinions, sentiments, perceptions, etc., to help develop tools in multiple situations to allow users’ interaction with applications, products, and services^40–42^. This is the possibility explored in this study, in which users are required to respond to a self-report standardized questionnaire that can be linked to smartphone dependency.

To have a precise notation, *x*^*(i)*^ denotes the input variables arranged as an *n*-dimension vector, also known as features, while *y*^*(i)*^ indicates the output or target variable (i.e., the predicting variable). The pair (*x*^*(i)*^*,y*^*(i)*^) is a training example. The dataset containing the information from *m* training examples {(*x*^*(i)*^*,y*^*(i)*^)}; *i* = *1… m*, is known as the training set. Typically, **X** and **Y** are used to denote the space representations of the input and output variables, respectively. When a classification problem is approached, the variables in the **Y** space take discrete values corresponding to the classes or categories defined in the learning problem. For the specific problem addressed in this work, *y* ∈ {0, 1}, where a value *y* = 0 has been defined to indicate a person with a negative diagnosis, whereas *y* = 1 indicates a person with a positive diagnosis of smartphone dependency.

A supervised learning problem estimates a function h_ɵ_(*x*): **X** → **Y**, such that given an input *x,* h_ɵ_(*x*) predicts the *y* value. The function h_ɵ_(*x*) is also known as the hypothesis function.

Several approaches have been applied to define the h_ɵ_ (*x*) function. From classical approaches such as logistic regression^43^, Support vector machines (SVM) with polynomial and Radial Basis Functions (RBF) kernels, which is considered a discriminative approach^44^, Decision tree^45^, and Random forest^46^, to modern approaches based on deep learning (DL) such as multilayer perceptron (MLP)^33^, and tabular data such as TabNet^47^, as is the particular case of the present study. A detailed description of previously mentioned techniques is out of the scope of this paper.

Deep learning techniques are well known for their performance when solving problems related to images, audio, and text^25,26^. One of the shortcomings of training a deep learning model is having sufficient data for a proper parameter estimation^26^. Some approaches include transiently modifying the output to fit the requirements and then fine-tuning learning, where a previously trained model can be applied^25^. However, in this work, the amount of data was relatively limited to infer that a deep neural network would be adequately trained; neither three are pre-trained models of adjacent problems so that transfer learning can be used. Hence, classical machine learning techniques are expected.

### System validation

The assisted diagnosis process using automated systems is imperfect. The result obtained from a classification system represents a probability rather than a correct answer with irrefutable certainty. Different diagnostic measures are thus employed to verify and assure that the results are repeatable and to validate the ability of a system to identify the presence or absence of disease.

In particular, random cross-validation (tenfold) was used in these experiments. The available data were used for data training (70%), and the remaining data (30%) to test the proposed model^33^. It is important to note that the folds were randomly assembled using a shuffle-split methodology in its stratified version to guarantee a proportional distribution in each set^34^. Each classification approach was evaluated using logistic regression, support vector machine, decision tree, random forest, multilayer perceptron, and TabNet. For assessing the performance of each model, diagnostic measures such as sensitivity, specificity, accuracy, and precision are used. Additionally, the area under the curve (AUC) of the receiver operating characteristics (ROC) was determined for each model^48,49^.

TP = true positive

TN = true negative

FP = false positive

FN = false negative

1 $$\mathrm{Accuracy }=\frac{TP+TN}{TP+TN+FP+FN}$$

2 $$\mathrm{Specificity }=\frac{TN}{TN+FP}$$

3 $$\mathrm{Sensitivity }=\frac{TP}{TN+FN}$$

4 $$\mathrm{Precision }=\frac{TP}{TP+FP}$$

## Results

The data analyses indicated that 70% of the participants presented smartphone dependence. Initially, a preliminary analysis was conducted to identify variables with a more prominent relationship with the response variable. Hence, the chi-square test for categorical variables and the odds ratio (OR) for dichotomous qualitative variables were applied. According to this analysis, the following variables were identified as related to smartphone dependency in students: (i) Academic program; (ii) school; (iii) marital status; (iv) socioeconomic status; (v) Is it possible to express oneself in the family? (vi) May the student be identified as not having a smartphone? (vii) Arguments about spending much time with a smartphone; (viii) residence area; (ix) the type of access to the network; (x) most used space; (xi) time of acquisition; (xii) average use time per day; (xiii) The posture you use when interacting with the phone: sitting on the floor, lying on the side, lying on the back; (xiv) the amount of time with body discomfort; and (xv) duration of each episode of wrist discomfort.

Table 1 shows the discriminated results for each variable. The risk factors are presented, and the variables and their corresponding sub-categories are indicated. The frequency and percentage of students classified as having dependency (cases) are also shown.

**Table 1:** Qualitative variables: university students with and without smartphone dependency.

Risk factors	Variables	Cases	Control	*p*-value
**Sociodemographic**	**Academic program**			
	Administration	42 (66.7)	21(33.3)	0.000
	Bioengineer	36(60.0)	24(40.0)	
	Accountancy	27(42.9)	36(57.1)	
	Law	201(68.1)	94(31.9)	
	Economics	29(46.0)	34(54.0)	
	Nursing	41(59.4)	28(40.6)	
	Finance	21(33.3)	42(66.7)	
	Physiotherapy	44(89.8)	5(10.2)	
	Speech Therapy	29(100.0)	0(0.0)	
	Industrial Engineering	60(100.0)	0(0.0)	
	Electronic Engineering	37(61.7)	23(38.3)	
	Engineering Energy	60(100.0)	0(0.0)	
	System Engineering	60(100.0)	0(0.0)	
	Surgical Instrumentation	21(60.0)	14(40.0)	
	Medicine	47(83.9)	9(16.1)	
	Marketing	31(64.6)	17(35.4)	
	Dentistry	37(90.2)	4(9.8)	
	Psychology	20(74.1)	7(25.9)	
	Respiratory Therapy	20(74.1)	7(25.9)	
	**Faculty**			
	Health	259(77.8)	74(22.2)	0.000
	Economic Sciences	150(50.0)	150(50.0)	
	Engineering	253(84.3)	47(15.7)	
	Law	201(68.1)	94(31.9)	
	**Civil status**			
	Single	737(72.0)	287(28.0)	0.021
	Married	48(66.7)	24(33.3)	
	Separated	8(53.3)	7(46.7)	
	Divorced	3(100.0)	0(0.0)	
	Widowed	1(100.0)	0(0.0)	
	Cohabitation	66(58.4)	47(41.6)	
	**Socioeconomic stratification (Lower class: 1—Upper class: 6)**			
	1	60(63.2)	35(36.8)	0.000
	2	167(55.5)	134(44.5)	
	3	367(74.1)	128(25.9)	
	4	159(78.3)	44(21.7)	
	5	96(82.8)	20(17.2)	
	6	14(77.8)	4(22.2)	
**Interpersonal**	**Who do you live with?**			
	Both parents	255(67.3)	124(32.7)	0.001
	Mother/father	153(72.2)	59(27.8)	
	Another family member	244(78.5)	67(21.5)	
	Friend	75(72.1)	29(27.9)	
	Partner	75(61.0)	48(39.0)	
	Alone	61(61.6)	38(38.4)	
	**Can you be discriminated for not owning a smartphone?**			
	No	537((66.5)	270(33.5)	0.000
	A little	139(74.7)	47(25.3)	
	Some	112(81.2)	26(18.8)	
	A lot	61(82.4)	13(17.6)	
	Extremely	14(60.9)	9(39.1)	
	**Arguments for spending too much time on the smartphone**			
	No	496(65.0)	267(35.0)	0.000
	A little	158(78.6)	43(21.4)	
	Some	110(76.9)	33(23.1)	
	A lot	74(80.4)	18(19.6)	
	Extreme	25(86.2)	4(13.8)	
	**Are there any rules for the smartphone use at home?**			
	No	243(58.1)	175(41.9)	0.000
	A little	414(74.3)	143(25.7)	
	Some	102(79.1)	27(20.9)	
	A lot	74(82.2)	16(17.8)	
	Extreme	30(88.2)	4(11.8)	
**Context sensitive**	**Type of internet connection**			
	Mobile data	432(65.3)	230(34.7)	0.000
	Wifi	422(76.0)	133(24.0)	
	Both	9(81.8)	2(18.2)	
	**Places of more smartphone use**			
	Home	430(66.3)	219(33.7)	0.004
	University	379(74.3)	131(25.7)	
	Shopping malls	54(78.3)	15(21.7)	
**Related to the mobile device**	**Time since you acquired your first cell phone**			
	Less than 6 months	42(80.8)	10(19.2)	0.000
	From 6 months to 1 year	250(75.1)	83(24.9)	
	1–3 years	87(60.4)	57(39.6)	
	3–6 years	140(59.6)	95(40.4)	
	More than 6 years	343(74.1)	120(25.9)	
	**Average time of use per day**			
	Less than an hour	35(61.4)	22(38.6)	0.000
	1–3 h	173(58.4)	123(41.6)	
	3–6 h	205(65.9)	106(34.1)	
	More than 6 h	449(79.8)	114(20.2)	
**Physical load**	**Do you use your smartphone sitting on the floor?**			
	Less than an hour	496(66.3)	252(33.7)	0.001
	1–3 h	339(77.2)	100(22.8)	
	3–6 h	24(72.7)	9(27.3)	
	More than 6 h	4(50.0)	4(50.0)	
	**Do you use your smartphone lying on one side?**			
	Less than an hour	495(64.2)	276(35.8)	0.000
	1–3 h	311(80.2)	77(19.8)	
	3–6 h	46(86.8)	7(13.2)	
	More than 6 h	11(68.8)	5(31.3)	
	**Do you use your smartphone lying on your back?**			
	Less than an hour	394(63.4)	227(36.6)	0.000
	1–3 h	371(77.8)	106(22.2)	
	3–6 h	75(78.1)	21(21.9)	
	More than 6 h	23(67.6)	11(32.4)	
	**How long have you had any discomfort?**			
	Less than a month	532(65.8)	277(34.2)	0.000
	Between 2 and 3 months	133(79.6)	34(20.4)	
	Between 4 and 6 months	81(78.6)	22(21.4)	
	Between 7 and 9 months	30(83.3)	6(16.7)	
	Between 10 and 12 months	87(77.0)	26(23.0)	
	**Duration of each wrist episode**			
	Less than an hour	730(68.8)	331(31.2)	0.014
	Between 1 and 24 h	80(82.5)	17(17.5)	
	Between 1 and 7 days	30(85.7)	5(14.3)	
	Between 1 and 4 weeks	9(69.2)	4(30.8)	
	More than a month	14(63.6)	8(36.4)	

The responses associated with the identification of musculoskeletal discomforts indicated the wrist as the body area with the highest risk factor (OR = 1.93, CI 95% = 1.47–2.54)). The neck, shoulder, back, and elbow regions showed similar risk factors (OR = 1.42, 1.62, 1.88, and 1.89, respectively). The results are summarized in Table 2.

**Table 2:** Bivariate analysis. Discomfort in undergraduate students with and without smartphone dependency.

Significative variables	Cases n	Controls n	OR (CR95%)
**Do you experience any discomfort in**			
Neck			
Yes	502	180	1.429 (1.118–1.827)
No	361	185	
Shoulder			
Yes	305	92	1.622 (1.233–2.134)
No	558	273	
Back			
Yes	347	96	1.884 (1.439–2.468
No	516	269	
Elbow			
Yes	163	40	1.892 (1.307–2.739)
No	700	325	
Wrist			
Yes	341	92	1.938 (1.476–2.547)
No	522	273	

Table 3 shows the results for the discomfort in the previous 12 months according to smartphone dependency. The results found the elbow (OR = 1.45) and shoulder (OR = 1.69) with the highest risk for discomfort, while the back area with the lowest.

**Table 3:** Bivariate analysis. University students experiencing discomfort in the last 12 months with and without dependency on the smartphone.

Significant variables	Cases n	Controls n	OR (CR95%)
**Discomfort during the last 12 months**			
Neck			
Yes	399	127	1.611 (1.251–2.077)
No	464	238	
Shoulder			
Yes	238	67	1.694 (1.250–2.296)
No	625	298	
Back			
Yes	309	101	1.458 (1.115–1.906)
No	554	264	
Elbow			
Yes	149	40	1.696 (1.168–2.462)
No	714	325	
Wrist			
Yes	580	280	1.607 (1.213–2.129)
No	283	85	

### Machine learning based prediction system

All the significant variables from the different models performed were included. A total of 31 variables related to smartphone dependence were identified. Table 4 shows the results for all classifiers in which the accuracy, specificity, sensitivity, precision, and area of the ROC curve of five diagnostic measures are presented. For the random forest, *n_e* is the number of estimators or trees in the forest. For SVM *C* is the regularization parameter, *γ* is the kernel coefficient for both polynomial and radial basis functions, and *d* is the degree of the polynomial kernel. In the case of the multilayer perceptron, we use a DNN with six hidden layers with 50, 50, 50, 20, 20, and 10 neurons using *relu* activation functions connected to an output layer with one single neuron using a *sigmoidal* activation function.

**Table 4:** Predictive Performance of the models.

Classifier	Accuracy	Sensitivity	Specificity	Precision	AUC
Decision tree	70.5 ± 2.0	78.9 ± 2.9	50.1 ± 2.7	79.2 ± 0.9	0.639 ± 0.01
Logistic regression	76.4 ± 1.5	91.1 ± 1.4	39.7 ± 3.4	78.4 ± 1.0	0.721 ± 0.02
**Random forest**					
*n_e* = *20*	76.6 ± 1.3	91.4 ± 0.7	40.6 ± 4.7	78.8 ± 1.2	0.731 ± 0.03
**SVM poly**					
*C* = *1, γ* = *5, d* = *2*	76.4 ± 1.2	93.1 ± 0.5	38.8 ± 3.9	77.9 ± 1.0	0.729 ± 0.01
**SVM rbf**					
*C* = *10, γ* = *3*	77.2 ± 1.3	92.2 ± 0.9	39.7 ± 3.6	78.7 ± 1.0	0.729 ± 0.01
Multilayer perceptron	73.6 ± 2.3	94.7 ± 5.2	22.7 ± 8.0	75.04 ± 3.1	0.720 ± 0.02
TabNet	67.4 ± 1.3	86.4 ± 5.3	21.3 ± 8.0	72.8 ± 2.2	0.611 ± 0.02

Differences were observed among the methods under study, considering the metrics to assess their performance. For example, the TabNet model and the decision tree have the lowest overall rates; however, the decision tree presented the highest specificity value, above 50%. In contrast, for logistic regression, random forest, and both support vector machine approaches, better sensitivity rates were achieved (above 91%), but specificity was significantly reduced (below 41%). As expected, neither the TabNet model nor the multilayer perceptron performed better than the classical approaches.

**Figure 3:**
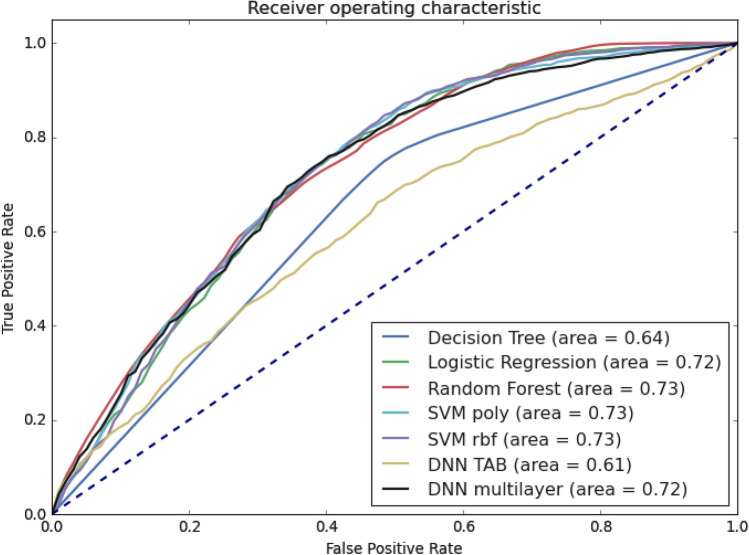
Receiver Operating Characteristic Curve (ROC curve) for all classification systems.

To perform a global evaluation for each classifier, the AUC of the ROC curve was determined (Fig. 3). It was observed that the classifier with the lowest performance was the TabNet model, followed by the decision tree. On the other hand, the similar AUC of the five models (AUC ~ 0.72) makes it challenging to determine which approach offers the best performance. Overall, considering the model's simplicity, the number of parameters, and the performance achieved by the logistic regression classification approach, such an approach is a suitable predictive model for the task at hand. However, the SVM or random forest classifiers constitute attractive alternatives, given that these approaches have comparable high performances.

It is worth mentioning that a highly sensitive system can correctly identify participants where smartphone dependency is suspected. Hence, self-reported information gathered through standardized questionnaires contains discriminative features to train predictive models. However, the perceptual and subjective nature of the information can also hamper the potential of predictive models. This may be the reason for achieving low specificity. In the early stages of a diagnosis, it is helpful to include the assessment of multiple professionals to reject or confirm dependency. It would be necessary to include objective measurements to improve the system’s prediction capabilities in future works.

## Discussion

The classification models yielded satisfactory smartphone dependency predictions. Likewise, a relationship between university students with and without smartphone dependency and multiple risk factors was found, which should motivate establishing high-priority preventive actions. The results indicate that student enrollment was significantly correlated with smartphone dependency, and an important prevalence was identified, especially among engineering (84.3%), health (77.8%), law (68.1%), and economic sciences students (50.0%). Similar results have been reported, although the highest dependency rate was identified in the medical academic program^50^.

Marital status (72%) was related to smartphone dependency, which is in line with previous studies^51–54^. However, being single cannot be included as a risk factor. It can be hypothesized that being involved in a romantic relationship may reduce smartphone users’ time. Nevertheless, this is a factor that requires additional analysis.

The high-income socioeconomic stratification was also meaningful for smartphone users, as it facilitates access to new technology, gadgets, pay-per-use applications, etc.^52,53,55^. Our data corroborate previous reports that high family income is more likely to develop smartphone dependency^17^. In addition, young students may feel discriminated against for not having a cell phone and not satisfying a communication prerequisite to belong to a particular social group. Cellphone ownership is highly relevant in today’s society, where social networks are at the core of personal and social relationships. It might have also accelerated the first cell phone acquisition, as dependency is more pronounced (74.1%) in those who used it for the first time more than six years ago. Others have also reported a similar dependence (77.5%)^56^. Further investigations are necessary to explore the causes of its acquisition and excessive use.

Adverse domestic situations can also be a predictor related to smartphone dependency^57^. It has been shown that students who reported domestic conflict or adversities (e.g., parent alcohol and drug use, mental health, incarceration, suicide, intimate partner violence, separation/divorce, and homelessness) are also more likely to have problematic/addictive smartphone use. A strong association between household dysfunction and psychological and behavioral health issues was reported. However, this association requires further research to explain this association further.

A significant difference was found between those who access the internet by paying for data packages and illimited access. Having internet access with no limitations facilitates surfing the internet, making video calls, gaming, sending text messages anytime, etc. The result showed that having a data plan increases the probability of developing smartphone dependency by 50%, as the number of hours is also likely to be greater than others with more limited access.

The amount of time spent using cell phones is also a strong indicator of dependence. In this study, the participants with smartphone addiction reported periods of usage longer than 6 h. It has been reported that the likelihood of developing smartphone addiction is proportional to the number of hours of use (3–4 h: OR = 5.79; 5–6 h: OR = 10.78)^17^. Indeed, the risk almost doubled for those using the device for 5–6 h compared to those with fewer hours (i.e., 3–4 h per day)^58^.

Sitting was the most predominant posture while using a smartphone (66.3%), despite the short period it was sustained (i.e., less than an hour). It may explain why the wrist and the neck areas showed the largest prevalence (OR. 1.93 and 1.42, respectively). It has been reported that office workers with excessive smartphone use are approximately six times more likely to have neck pain^59^. It reinforces that smartphone dependency is highly associated with neck pain. Nonetheless, the prevalence was lower than reported by Derakhshanrad and colleagues^59^. There can be multiple reasons for this difference, including the location, target population, and instrument applied. In this study, university students with smartphone dependency reported discomfort or musculoskeletal symptoms for less than one month (n = 532, 65.8%). Hence, the presence and duration of musculoskeletal discomfort in the last 12 months contribute to the prediction of smartphone dependency.

The variables used in the model show that sociodemographic characteristics determine a level of smartphone dependency. However, the age and gender variables must be ruled out. For instance, Nikhita and collaboratives reported that female users had a higher prevalence^60^, while Matoza-Báez and colleagues^61^ showed a higher prevalence of male users. The age of more than 90% of our participants ranged between 18 and 32, and a more comprehensive range is required to discard age as an explanatory factor.

This is a cross-sectional analysis, and longitudinal studies are required before establishing a cause-effect relationship. The inclusion and analysis of variables related to academic performance, mental health, and sleep disorders may be considered for future studies. Although the number of participants included in the present study is not trivial, the amount of data affects the training process of the models, and it remains an open problem to address in future studies, including deep learning techniques. Once risk factors and variables related to smartphone dependency are identified, it is essential to mention that strategies to reduce these risks and adverse effects are paramount for society. It should involve a multidisciplinary approach. Campaigns to raise awareness about the negative consequences of physical and mental health and how to address these problems or where people can find professional advice may constitute a relevant strategy to counteract the adverse impacts of overusing technology.

## Conclusions

Smartphones are ubiquitous and part of our daily life. The adverse effects of excessive use of smartphones are concerning, as dependency is becoming a public health problem requiring special attention due to its consequences on physical and mental health. Machine learning helped identify several dependency factors while using a large number of independent variables. The support vector machine and random forest presented the highest prediction precision for smartphone dependency, obtained through the stratified-k-fold cross-validation technique. The variable selection is more critical than the choice of a specific model itself.

This study shows that self-reported information obtained using standardized questionnaires contains discriminative information to predict smartphone dependency using data-driven models. These results open doors for future studies aiming to reduce the adverse effects of overusing mobile devices. In many cases, a correct assessment of dependency levels and the corrective actions to be taken require the intervention of experienced health professionals. This is not always possible in the early stages, while late interventions can be costly and may bring adverse effects. Further research in this area is still required, as the perceptual and subjective nature of the information may hamper the potential of predictive models. For future work, it is necessary to introduce objective measures. Using electronics to measure physiological activity can add important information instead of subjective self-reported variables.

## Data Availability

Datasets analyzed during the current research are available to the corresponding author upon reasonable request.

## Abbreviations

MD: Mobile device
SDT: Smartphone dependency test
OR: Odds ratio
MSD: Musculoskeletal disorder
ROC: Receiver operating characteristic
SVM: Support vector machine
PSU: Problematic/Addictive smartphone use
